# A niche for axial stem cells - A cellular perspective in amniotes

**DOI:** 10.1016/j.ydbio.2022.06.015

**Published:** 2022-10

**Authors:** Tatiana Solovieva, Valerie Wilson, Claudio D. Stern

**Affiliations:** a Department of Cell and Developmental Biology, University College London, UK; b Centre for Regenerative Medicine, The University of Edinburgh, UK

## Abstract

The head-tail axis in birds and mammals develops from a growth zone in the tail-end, which contains the node. This growth zone then forms the tailbud. Labelling experiments have shown that while many cells leave the node and tailbud to contribute to axial (notochord, floorplate) and paraxial (somite) structures, some cells remain resident in the node and tailbud. Could these cells be resident axial stem cells? If so, do the node and tailbud represent an instructive stem cell niche that specifies and maintains these stem cells? Serial transplantation and single cell labelling studies support the existence of self-renewing stem cells and heterotopic transplantations suggest that the node can instruct such self-renewing behaviour. However, only single cell manipulations can reveal whether self-renewing behaviour occurs at the level of a cell population (asymmetric or symmetric cell divisions) or at the level of single cells (asymmetric divisions only). We combine data on resident cells in the node and tailbud and review it in the context of axial development in chick and mouse, summarising our current understanding of axial stem cells and their niche and highlighting future directions of interest.

## Highlights

• The head-tail axis in birds and mammals arises from a caudal growth zone, containing the node.
• This growth zone then forms the tailbud.
• This review examines the evidence that the node and tailbud contain resident axial stem cells.
• We combine data on resident cells in the node and tailbud and discuss for chick and mouse.
• We highlight future directions of interest.

## Graphical abstract

**Figure:**
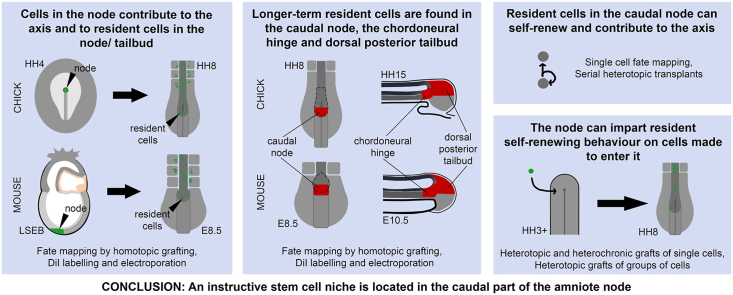

## 1 Introduction

Development of the head-to-tail (rostro-caudal) axis in birds and mammals occurs from a growth zone at the tail end. The node, and later the tailbud, contribute to the growth zone and are thought to contain resident stem or long-term progenitor cells that remain within the node and tailbud while their progeny contributes to axial and paraxial structures. However, when it comes to evidence for such stem and progenitor cells, there are some discrepancies between results and conclusions reported in the literature. Here we review evidence for the existence of such resident cells in avian and mammalian models throughout axial elongation, discuss the importance of studying cell behaviour at the single cell level and highlight some unresolved questions.

## 2 Role of the node

The node region has two important, conserved signalling functions: as an organizer of the nervous system (Spemann, 1921; Spemann and Mangold, 1924; Waddington, 1934, 1937) and a role in patterning (“dorsalization”) of the adjacent mesoderm (Dale and Slack, 1987; Nicolet, 1970; Streit and Stern, 1999). Another well characterized role of the node is its cellular contribution to axial (notochord, floorplate) and paraxial (somite) tissues and definitive gut endoderm (Rosenquist, 1966, 1983; Schoenwolf et al., 1992; Selleck and Stern, 1991; Spratt, 1955). More recently it has also been shown to act as a niche for resident axial stem cells in chick (Solovieva et al., 2022), a novel role which we will discuss in more detail.

### 2.1 The node and tailbud through axial elongation

Rather than defining a cell population, Hensen's node encompasses a region at the tip of the primitive streak in the developing embryo from the end of gastrulation through to the onset of secondary neurulation (Izpisúa-Belmonte et al., 1993; Joubin and Stern, 1999). Hensen's node, or ‘Knoten’, in German, was first given its name in mammals by Viktor Hensen in 1876 from his studies of development in rabbit and guinea pig embryos (Hensen, 1876; Viebahn, 2001), and has since been used to refer to the equivalent structure across amniotes. According to Hensen, ‘Hensen's node’ defines only the morphological node, in which three germ layers are fused (Hensen, 1876; Leikola, 1976).

**Fig. 1:**
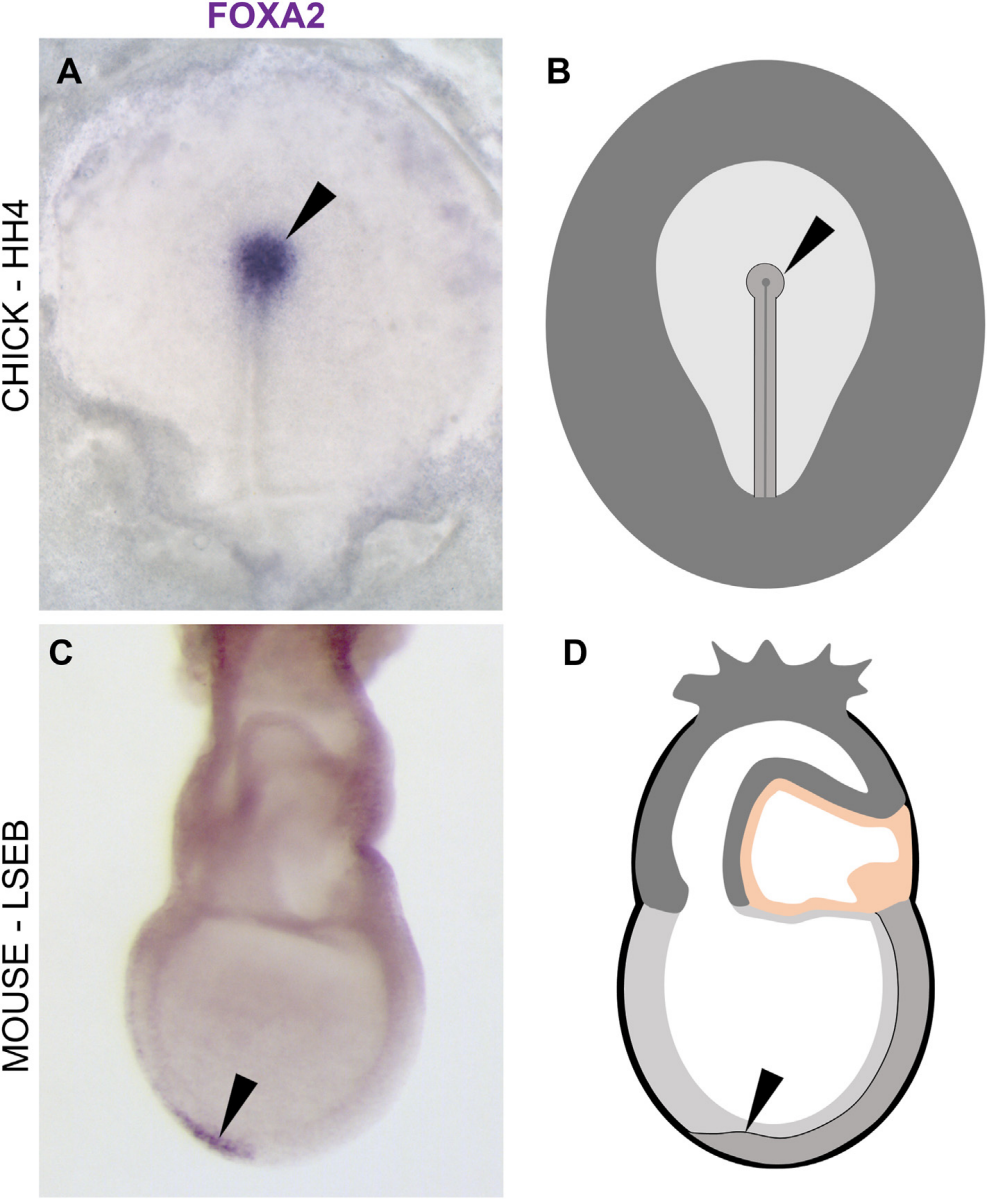
**Hensen's node in chick and mouse.***In situ* hybridization against FOXA2 (a molecular marker for node) in both chick (A–B) and mouse (C–D) shown at the stages when the morphological node becomes evident alongside schematics of the embryos. Black arrows point to the node. HH4, Hamburger Hamilton stage 4; LSEB, Late Streak Early Bud stage.

In chick, the morphological node or ‘Hensen's node’ (Fig. 1), is visible from HH4 (∼18 ​h incubation) (Hamburger and Hamilton, 1951), from Theiler stage 10b in mouse (∼E7) (Theiler, 1972) and from Carnegie stage 7 in humans (∼15–17 days gestation) (O'Rahilly, 1973, 1987; Rulle et al., 2018). It is important to note that the appearance of the morphological node does not necessarily correspond to the timing of the region's signalling functions, for example the neural inducing ability of the node region begins at HH3+ in chick, (around 14 ​h incubation) a few hours before the morphological node becomes visible. The cellular composition, shape and position of this region changes gradually through development and ultimately becomes incorporated into the tailbud, which continues to lay down axial and paraxial structures until the end of axial elongation (Cambray and Wilson, 2002, 2007; Catala et al., 1995; Knezevic et al., 1998). The ‘tailbud stage’, the point in development when the postanal tail begins growing, begins from around 22 somites in chick (HH14, 50–53 ​h of incubation) (Criley, 1969; Schoenwolf, 1979) around 30 somites in mouse (Theiler stage 16, E9.5-10) (Schoenwolf, 1984) and around 25 somites in human (Carnegie stage 12, ∼26–30 days gestation) (Müller and O'Rahilly, 1987). The tailbud stage continues until the end of axis elongation when all 52 somite pairs have been formed in chick (HH22 after 3.5–4 days incubation) (Sanders et al., 1986), up to 65 somite pairs in mouse (Theiler stage 23, E15) (Theiler, 1972) and 38–39 somite pairs in humans (Carnegie stage 14–16, 31–42 days gestation) (O'Rahilly and Müller, 2003) (summarised in Table 1).

**Table 1:** **Developmental events during axial elongation across chick, mouse and human embryos. ‘**HH’ represents Hamburger Hamilton stages of chick development (Hamburger and Hamilton, 1951). ‘Theiler’ refers to mouse developmental stages (Theiler, 1972). ‘Carnegie’ refers to human developmental stages (Müller and O'Rahilly, 1987; O'Rahilly, 1973, 1987; O'Rahilly and Müller, 2003).

Developmental event	Organism	No. of somites	Organism-specific stage	Developmental time
**Hensen's node appears**	Chick	0	HH4	18 ​h
	Mouse	0	Theiler 10b	7 days
	Human	0	Carnegie 7	15–17 days
**Tailbud appears**	Chick	22	HH14	50–53 ​h
	Mouse	30	Theiler 16	9.5–10 days
	Human	25	Carnegie 12	26–30 days
**Axial elongation ends**	Chick	52	HH22	3.5–4 days
	Mouse	65	Theiler 23	15 days
	Human	38–39	Carnegie 14-16	31–42 days

### 2.2 Cell dynamics in the node

**Fig. 2:**
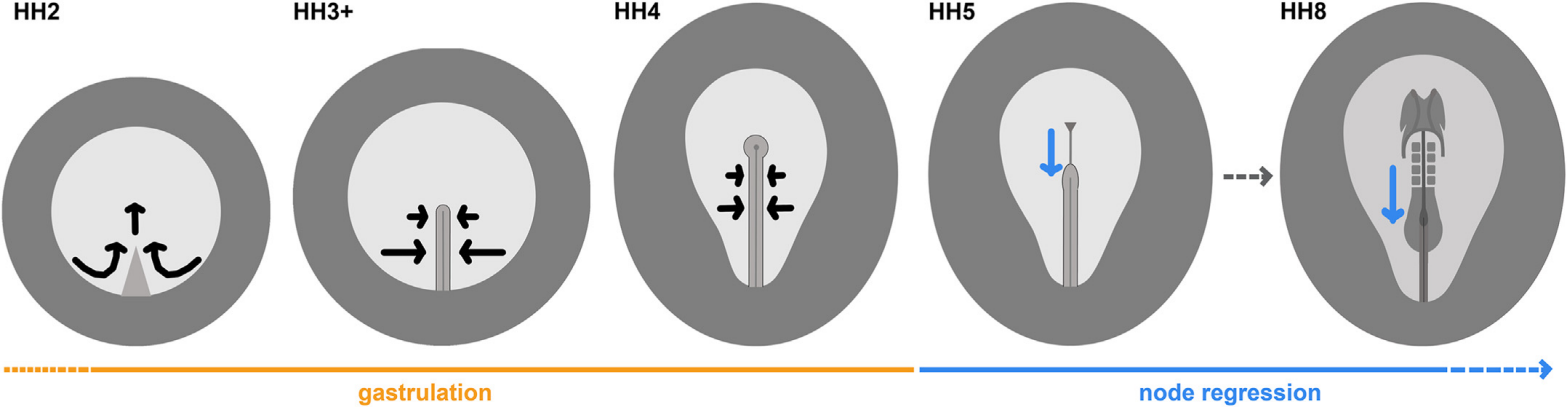
**Early node development in chick.** Development of the chick embryo from the beginning of gastrulation (HH2), through the appearance of the morphological node (HH4), to the beginning of node regression from HH5. ‘Regressing node’ stages continue past HH8 until HH14 when the node incorporates into the tailbud. Black arrows show the direction of epiblast cell movements during gastrulation. Blue arrows show regression of node from the previous developmental stage pictured.

The node is formed by the coming together of two distinct cell populations (Streit et al., 2000): the ‘posterior deep’ cells which are contained within the tip of the elongating primitive streak (Izpisúa-Belmonte et al., 1993), and the ‘central epiblast’ which moves from a posterior to a central position in the blastoderm before primitive streak formation (Hatada and Stern, 1994). Cells also enter the anterior primitive streak and node from the neighbouring epiblast during gastrulation (“primitive streak stages”, stages HH2-4 in chick and early-to mid-streak in mouse), ingressing through in a dorsal to ventral manner before leaving and contributing to endodermal, mesodermal (notochord and somites) and neural (medial floorplate) structures (Rosenquist, 1966, 1983; Schoenwolf et al., 1992; Selleck and Stern, 1991; Spratt, 1955) (Fig. 2). While passing through the node, cells temporarily gain a node-like molecular signature, visible by temporary expression of node markers such as ADMP (anti-dorsalizing morphogenetic protein) and CHRD (chordin) (Joubin and Stern, 1999). Following gastrulation, while cell ingression continues along the more posterior primitive streak, ingression ceases at the level of the node (HH4+ in chick and following late-streak stage in mouse), which begins to regress in a caudal direction (Spratt, 1947) while simultaneously laying down axial (notochord, floorplate) and paraxial (somites) structures (Fig. 2). The contribution of the node and anterior primitive streak to the definitive (gut) endoderm ceases by HH4, when all of the precursors have ingressed into deeper layers (Gallera and Nicolet, 1969; Kimura et al., 2006; Rosenquist, 1966). From these stages to the formation of the tailbud (HH14-16 in chick and E9.5-10 in mouse), we will refer to the node as the ‘regressing node’.

### 2.3 Presence of resident cells

**Fig. 3:**
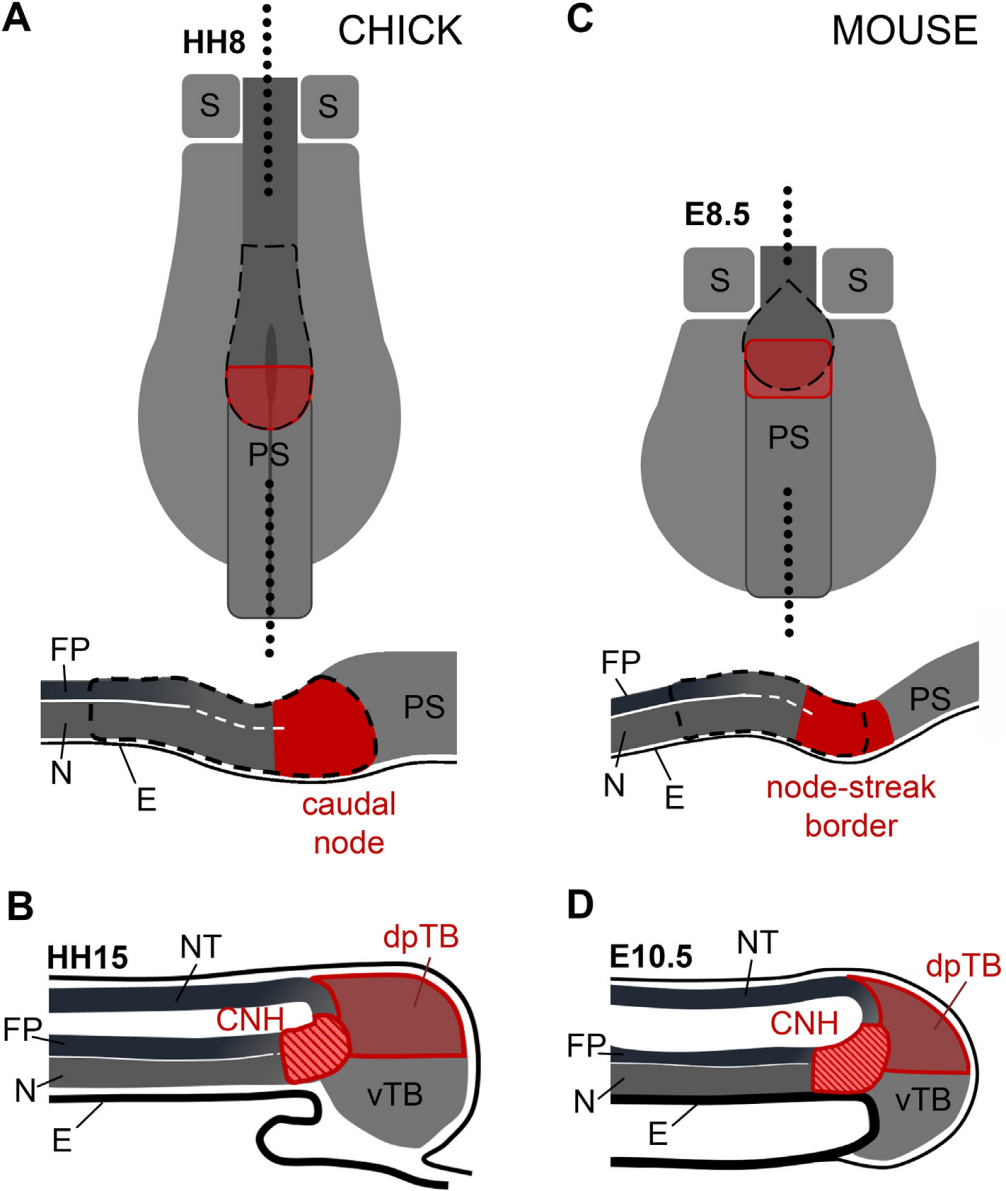
**Location of resident cells in the node and tailbud.** Caudal part of chick (A–B) and mouse (C–D) embryos shown during regressing node stages (A, C) and tailbud stages (B, D). All areas in shades of red show regions containing cells with resident behaviour, as shown through homotopic grafts (Cambray and Wilson, 2007; Catala et al., 1995, 1996; McGrew et al., 2008), electroporation (Mathis et al., 2001) or DiI labelling (Knezevic et al., 1998; Selleck and Stern, 1991; Solovieva et al., 2022; Wilson and Beddington, 1996). The location of resident cells in the mouse tailbud was inferred from heterochronic grafts (Cambray and Wilson, 2002; McGrew et al., 2008). Dashed red highlights regions containing long-term self-renewing stem cells (at least at the population level), as suggested by serial transplants of cell populations (Cambray and Wilson, 2002; McGrew et al., 2008). Solid red marks regions containing an instructive stem cell niche, as suggested by heterotopic grafts of single and/or groups of cells (Cambray and Wilson, 2007; Solovieva et al., 2022), and cell-cycle analysis (Bellomo et al., 1996; Solovieva et al., 2022; Wymeersch et al., 2019). Black dashed lines outline the node. The level of the sagittal sections through is shown with black dotted lines in 'A' and 'C'. Only the most caudal pair of somites is shown in ‘A’ and ‘C’. CNH ​= ​chordoneural hinge; dpTB ​= ​dorsal posterior tailbud; vTB, ventral tailbud; S, somite; PS, primitive streak; FP, floorplate; N, notochord; E, endoderm; NT, neural tube.

While many cells leave the node to give rise to axial and paraxial structures, some cells remain resident within the node, regressing node and tailbud; we will refer to these as ‘resident cells’ or cells with ‘resident behaviour’. From the collective point of view of the cell population that makes up these structures, resident behaviour in the node, regressing node and tailbud has been demonstrated through homotopic grafts (Cambray and Wilson, 2007; Catala et al., 1995, 1996; McGrew et al., 2008), electroporation (Mathis et al., 2001) and DiI labelling (Knezevic et al., 1998; Selleck and Stern, 1991; Solovieva et al., 2022; Wilson and Beddington, 1996). Tracking cells and their descendants from sub-regions of the regressing node and tailbud has revealed that longer term resident cells reside only in the posterior regions of the regressing node (Cambray and Wilson, 2007; Solovieva et al., 2022; Wymeersch et al., 2019), and in the chordoneural hinge (CNH) and dorsal part of the posterior tailbud (Cambray and Wilson, 2002; McGrew et al., 2008) (Fig. 3).

## 3 Are resident cells stem cells?

A stem cell is defined by its ability both to self-renew and to contribute to at least one differentiated cell type (Becker et al., 1963; Siminovitch et al., 1963; Till et al., 1964). One way to identify stem cells is therefore to look for self-renewal within the node. However, there are different mechanisms of self-renewal, each with different implications on environmental requirements.

### 3.1 Stem cell models

Two main mechanisms are known by which stem cells can self-renew. The first involves asymmetric division of individual stem cells, where one daughter self-renews and the other daughter differentiates, such as the germline stem cells (GSCs) in the *Drosophila* reproductive system (De Cuevas et al., 1998; Gonczy and DiNardo, 1996; Lin and Spradling, 1995). The second mechanism is through renewal at the population level, but individual cell divisions may be either asymmetric or symmetric. One example of this type of self-renewal is found at the base of the intestinal crypt where the behaviour of crypt base columnar (CBC) stem cells depends on their position within the crypt niche. While all CBC cells appear to be able to self-renew, cells that end up closer to the centre of the niche are reported to have a short-term advantage in self-renewal potential (Ritsma et al., 2014).

While renewal at the population level requires an external environment (or niche) to maintain the stem cells and facilitate differentiation in a subset of daughters, individual asymmetric divisions may be regulated either by the local environment or cell autonomously. An example of intrinsic regulation of asymmetrical self-renewal is through unequal segregation of cell fate determinants, as in *Drosophila* neuroblasts*,* where apical localisation of the ‘inscuteable’ protein is required for asymmetric cell division (Kraut et al., 1996; Schober et al., 1999; Wodarz et al., 1999). In contrast, requirement of an external niche for self-renewal at the single cell level include the testes and ovaries of *Drosophila,* where niche support cells are required to maintain the germline stem cells (Kawase et al., 2004; Kiger et al., 2001; López-Onieva et al., 2008; Tulina and Matunis, 2001; Xie and Spradling, 2000).

In the following sections we will discuss data from experiments involving the node, regressing node and tailbud and discuss whether they support self-renewal occurring at the level of a population or of individual cells, a combination of both, or neither.

### 3.2 Evidence from single cell labelling

Fate mapping of a single cell in the node has been performed in chick (Selleck and Stern, 1991) and mouse (Forlani et al., 2003; Lawson et al., 1991) primitive-streak-stage embryos using intracellular injection of lysine rhodamine dextran and horseradish peroxidase, respectively. In both organisms, following culture of the labelled embryos, multiple labelled cells were observed in the regressing node and node derivatives along the axis. This suggests that the single labelled cell had divided within the node and contributed progeny to the developing axis. Cells in the notochord and somites were sometimes observed in a periodic pattern of clusters. In such cases the fluorescence of rostral clusters was diluted compared to that of more caudal clusters, suggesting that cells in more rostral clusters had divided more and thus diluted the lysine rhodamine dextran. This is supported by the observation that rostral clusters appeared to contain twice the number of cells compared to the next more caudal cluster (Selleck and Stern, 1991, 1992). This pattern is most easily explained by a single self-renewing stem cell having been labelled in the node, which has then undergone regular asymmetric divisions where only one of the daughters at each division leaves the node and contributes to the notochord/somite, where it continues to divide. This suggests that asymmetric self-renewal can take place at the single cell level in the node.

Such periodic clusters also provide insight into cell cycle time of notochord versus somite progenitors in the node. For example, in the notochord clusters were separated by roughly 2-somite lengths, and clusters in the medial somites separated by roughly 5–6 somite lengths (Selleck and Stern, 1991, 1992). This pattern suggested that labelled progeny leave the node for the notochord every 3 ​h (2 x the 90 ​min it takes to make a somite pair), and progeny destined for the somites leave every 7.5–9 ​h (5 or 6 ​× ​90 ​min), showing a faster cycle time for notochord versus somite progenitors (Selleck and Stern, 1992).

### 3.3 Evidence from serial transplantation

One way to test for long-term self-renewal of cells in the node and tailbud is to perform heterochronic grafts into a younger host multiple times, to see if axial progenitors can contribute repeatedly to a given axial level. In mouse, homotopic GFP node grafts at E8.5 give rise to GFP labelled cells along the axis, and in the CNH and TBM by E10.5 (∼30–35 somites). These GFP-labelled CNH or TBM regions were heterochronically grafted into the node-streak border of ∼3–8 somite (E8.5) embryos and cultured up to ∼E10.5 before re-grafting into a second E8.5 host, if resident cells remained (Cambray and Wilson, 2002). The CNH contributed to the tailbud for two of these heterochronic transplants and also continued to contribute to notochord, somites and neural tube. In contrast, the TBM did not give rise to resident cells following heterochronic transplant into the node-streak border (Cambray and Wilson, 2002). Serial transplantations were also performed on the chick CNH (McGrew et al., 2008). The CNH was first labelled by homotopic grafting from a GFP donor at the 24–27 somite stage (HH15), cultured to 40–43 somites (HH20) and then re-grafted into a younger tailbud. Such serial transplants were performed three times and contributed to cells with resident behaviour in the tailbud at each transplantation (McGrew et al., 2008), similar to the results of mouse CNH serial transplants (Cambray and Wilson, 2002). Homotopic grafts of chick ventral tailbud did not contribute to the later tailbud, suggesting that the ventral tailbud does not contain resident cells (McGrew et al., 2008). However, serial transplants of dorsal posterior tailbud (dpTB) in chick were found to contribute to the tailbud, but only in the first heterochronic graft (McGrew et al., 2008), suggesting that it does not contain long term resident cells.

Serial transplantation of resident cells therefore suggests that cells in the CNH of the mouse and chick tailbud are able to contribute repeatedly both to appropriate axial structures and to cells with resident behaviour in the regressing node and tailbud. To date, this is probably the most convincing demonstration of long-term self-renewal, at least at cell population level (Fig. 3).

### 3.4 Axial positional information

Serial transplantation suggests that resident axial progenitors can contribute to all axial levels and that they can do so repeatedly. Does the molecular identity of these heterochronically grafted cells also adjust to the appropriate axial level?

When cells from a 24–27 somite chick embryo (HH15) are grafted into the younger node (4 somites, HH8), the molecular identity of graft derived cells appears to match that of their new rostro-caudal axial identity, as demonstrated by *in situ* hybridization for homeobox genes (McGrew et al., 2008). The expression of HOXA10*,* (expressed in the chick tailbud at HH15 but not at HH8), was lost in tailbud graft-derived cells when checked 8 ​h after grafting into the younger host, suggesting that the graft-derived cells had acquired a more rostral molecular signature. Furthermore, graft-derived cells contained HOXC8 protein (normally present at the level of the 22nd somite from ∼HH21) four days after the graft (McGrew et al., 2008).

This suggests that resident cells in the tailbud can adjust to their environment and acquire molecular signatures appropriate to their new axial level. However, this ability is not shared by all cells. Progenitors of paraxial mesoderm in the streak for example, do not appear to be able to gain a more rostral HOX identity as demonstrated through heterochronic grafts of streak tissue from an older embryo into a younger embryo, where the grafted tissue retains posterior Hox gene expression (Iimura and Pourquié, 2006). Furthermore, ectopic somites derived from posterior streak explants retain Hox codes corresponding to their original identity (Dias et al., 2014).

### 3.5 ‘Stem cells’ versus ‘progenitor cells’

When a cell self-renews, should its daughter have an identical molecular signature? The changing molecular signature of the node and tailbud during development as it lays down successively more caudal axial tissues raises the question of whether true self-renewal takes place in the node and tailbud. Even if a daughter were to remain in the regressing node/tailbud following a cell division, successive resident daughters might have different molecular signatures to one another. This is one reason why resident cells have sometimes been referred to as ‘progenitors’, rather than ‘stem cells’ (McGrew et al., 2008). Another view would be that molecular changes over time do not necessarily result in a change in potency. In support of this, heterotopic grafts with expression of appropriate HOX genes in donor cells (McGrew et al., 2008) and serial transplants with repeated contribution to appropriate axial structures (Cambray and Wilson, 2002; McGrew et al., 2008) demonstrate that molecular changes are not necessarily permanent and can be reset or ‘reprogrammed’ by changing the environment. This highlights that the gene expression profile does not necessarily predict the developmental potential of these cells and is only a snapshot of these cells at a point in time. The developmental potential of these cells and their ability to self-renew are consistent with them being true stem cells.

## 4 Is the node an instructive stem cell niche?

The existence of resident stem cells in the node and tailbud raises the question of where these cells acquire these properties. Are cells specified to self-renew before the node is formed and simply maintained in a permissive environment within the node, or are they instructed to acquire their characteristic behaviours by a niche within the node? In other words, is the node an instructive stem cell niche, defined by its ability to induce and maintain stem cell properties, as first described for the haematopoietic stem cell niche (Dexter et al., 1977; Schofield, 1978)?

### 4.1 Insights from manipulating cell populations

One way to ascertain whether an instructive stem cell niche is present in a given region is to design experiments that change the cell population in that region. For example, a suitable test might include addition of cells external to the candidate niche, followed by observation of the behaviour of the donor-derived cells in their new location.

During regressing node stages in chick and mouse, axial stem cells appear to reside in the caudal part of the regressing node (the axial paraxial hinge/node-streak border) where as a population, cells contribute to axial structures (notochord, ventral neural tube and/or somites) and to cells with resident behaviour that remain in the node/tailbud (Cambray and Wilson, 2007; Solovieva et al., 2022; Wymeersch et al., 2019) (Fig. 3). The more rostral part of the node which normally contributes only to axial tissues, when grafted to the caudal node, can contribute to both axis and CNH of the tailbud (Cambray and Wilson, 2007). Furthermore, caudal lateral epiblast adjacent to the node, after grafting to caudal node at E8.5, increases its contribution to CNH (Wymeersch et al., 2016). The environment of the caudal node therefore appears to impart behaviour that resembles self-renewal at the population level, although it does not necessarily imply self-renewal at the single cell level (Fig. 3).

At later developmental stages, the rostral part of the tailbud (CNH) and dorsal posterior tailbud is considered to be the site of long-term axial progenitors/stem cells (Cambray and Wilson, 2002; Catala et al., 1995; McGrew et al., 2008). Both of these regions normally contribute to cells with resident behaviour in the tailbud as axial development continues (Cambray and Wilson, 2002; Catala et al., 1995; McGrew et al., 2008). When the ventral tailbud, which does not contribute to resident cells, is grafted into either the dorsal posterior tailbud or the CNH, it still fails to contribute to cells with resident behaviour, and continues to contribute only to paraxial mesoderm, as it does from its usual position. Since the caudal node (node-streak border) and rostral tailbud (CNH) are considered to be topologically continuous with one another during development (Cambray and Wilson, 2007; Charrier et al., 1999), heterochronic grafting results from tailbud stages suggest that the environment of long-term axial progenitors changes over time, losing its ability to endow cells with self-renewal behaviour at tailbud stages. However, an alternative explanation could be that ventral tailbud cells are not competent to respond to the environment in the rostral tailbud (CNH) or in the dorsal posterior tailbud.

### 4.2 Considerations related to studies at the population level

Although heterotopic manipulations of cell populations can be used to test for instructive niche properties, it is not possible to assess dynamics at the level of a single cell and therefore any stem-cell like behaviour (self-renewal) can only be assessed at the population level. Furthermore, we do not yet know the size of putative niche(s) in the node, so a graft of multiple cells could potentially disrupt the activity of an existing niche. Some characterized niches contain as few as one stem or niche cell (Crittenden et al., 2006; Nystul and Spradling, 2007), therefore an introduced graft might create a ‘community effect’ (Gurdon, 1988) whereby the donor graft might behave as its own niche, or be too large to respond to the environment of a local host niche. One approach to resolve some of these issues is to perform heterotopic grafts with single donor cells, and to observe their behaviour in the host.

### 4.3 Insights from manipulating single cells

Such heterotopic single cell grafts have been performed in the chick to look for stem cell self-renewal at the single cell level (Solovieva et al., 2022). First, a group of anterior epiblast cells (from a GFP donor) that do not normally enter the node were made to do so by grafting just lateral to the tip of the streak/node (HH3+/4) from where they entered the node, driven by the morphogenetic movements of gastrulation. After culture to the ∼5 somite stage (HH8), graft derived cells were found to have contributed both to axial tissues (notochord and somites) as well as giving rise to cells with resident behaviour in the regressing node of the host. A single graft-derived (GFP positive) resident cell was then removed and grafted into the node of a second primitive-streak-stage (HH3+/4) host to check for self-renewal behaviour. After culture to the 5 somite stage (∼HH8), GFP cells were present in both node and axial midline of the second host, showing that the single grafted GFP cell had divided, contributed to the axis and self-renewed within the node (Solovieva et al., 2022). This indicates that the node can specify asymmetric self-renewing axial stem cells from cells that would not normally enter the node and thus represents an instructive stem cell niche (Fig. 3).

### 4.4 Structure of the niche

These results suggesting the existence of an instructive niche in the node raise questions about the structure of this niche: what is its composition, and does it change over time?

Questions that still remain are how many stem cells there are in the node and tailbud during axial development and whether this number changes over time. In the primitive-streak-stage chick node (∼1000 ​cells total), an estimate for the number of medial somite progenitors has been calculated (using cell cycle length and cell number per somite, along with an estimate of the number of cell divisions undergone by their precursors in the pre-somitic mesoderm) as just 64 ​cells (Selleck and Stern, 1991, 1992; Stern, 1996) (and unpublished observations). Although not the only progenitor type within the node, this corresponds to a very small proportion (∼6% of the cells) of the overall node.

Transcriptomic analysis of single cells with resident behaviour in the caudal part of the chick node revealed that these cells have high levels of G2/M phase cell cycle genes relative to most of their neighbours in the posterior part of the node suggesting that these cells are preparing to divide (Solovieva et al., 2022). Importantly, this enrichment of G2/M cell cycle genes is only present in a few cells of the caudal node region, suggesting that cells preparing to divide make up just a small part of the caudal node. Interestingly, BrdU staining in mouse suggests that most cells in the ventral part of the node are quiescent (Bellomo et al., 1996; Wymeersch et al., 2019), while both fast- and slow-cycling cells are present at the caudal extreme of the ventral node (the node-streak border). In contrast, cells in the dorsal node are generally fast-cycling. In chick, descendants of single GFP-positive cells grafted into the node have been observed in both dorsal and ventral parts of the caudal region of the node following self-renewal (Solovieva et al., 2022). Together, these observations suggest that stem cells comprise a small fraction of the node, are located in the caudal node region and cycle with different kinetics from the remainder of the node (Fig. 3).

## 5 Lifetime of the niche

For how long does the niche function to specify and maintain stem cells in the node and tailbud? As discussed in earlier sections heterotopic grafts of single cells into the node have shown that the node can act as an instructive stem cell niche from the primitive streak stage (Solovieva et al., 2022). However heterotopic grafts at tailbud stages have so far been inconclusive on the properties of the instructive stem cell niche. One alternative way to assess the lifetime of the niche is to look at the lifetime of resident cells.

### 5.1 Lifetime of resident cells

Resident cells have been identified in mouse and chick over a range of developmental stages (Cambray and Wilson, 2007; Catala et al., 1995, 1996; Forlani et al., 2003; Knezevic et al., 1998; Lawson et al., 1991; Mathis et al., 2001; McGrew et al., 2008; Selleck and Stern, 1991; Solovieva et al., 2022; Wilson and Beddington, 1996), but so far there have been no studies in which single cells have been mapped prospectively from the primitive streak stage to the end of axial elongation. It is therefore unclear whether the pool of resident cells remains the same throughout axial development (with clonal continuity between stem cells at all axial levels), or whether it constitutes two or more distinct pools (where there is no continuity between stem cells of rostral and caudal axial levels), a point which has been debated (Eloy-Trinquet et al., 1999; Nicolas et al., 1996; Roszko et al., 2007).

Retrospective clonal analysis using a rare, random recombination event to mark a single cell and its descendants in mouse suggests that long-term resident cells that contribute to the notochord, floor plate and somites at axial levels from head-to-tail exist from early primitive streak stage to tailbud stages at E10. 5 (∼35–43 somites) (Tzouanacou et al., 2009). It is important to note however, that in retrospective clonal analysis we cannot be sure where the labelled cell was located at the time of labelling or indeed the stage at which the labelling occurred, therefore we cannot be sure that a cell was resident in the node at the time of labelling. The pattern and frequency of clones contributing to neural tube and somites along the rostro-caudal axis suggests a loss of some resident progenitors from gastrula stages, and addition of ‘new’ resident progenitors between regressing node and tailbud stages (Tzouanacou et al., 2009), possibly derived from caudal lateral epiblast (Brown and Storey, 2000). The retrospective clonal analysis also hints that separate populations may produce notochord, floor plate and somites on the one hand, and neural tube and somites (elsewhere termed ‘neuromesodermal progenitors’) on the other. Collectively, this suggests the presence of some resident stem cells that exist from gastrula stage and contribute to all axial levels, with an addition of another pool of resident stem cells between regressing node and tailbud stages that contribute only to caudal parts of the axis.

## 6 Ending axial extension

Axial extension ends at ∼52 somite pairs in chick (Sanders et al., 1986) and ∼65 somite pairs in mouse (Theiler, 1972) (Table 1). What part do resident stem cells have in ending axial extension? There are three possible cellular mechanisms: either all resident stem cells leave the tailbud and differentiate, or the stem cells stop dividing, or they are eliminated through cell death.

### 6.1 Loss of resident cells from the tailbud

Loss of cells from the tailbud by loss of resident cell behaviour might occur through the loss of FGF signalling (Olivera-Martinez et al., 2012), which has been suggested to have a role in maintaining resident cell behaviour (Mathis et al., 2001). Loss of FGF signalling has also been attributed to an increase in retinoic acid within the tailbud just prior to the end of axis elongation (Cunningham et al., 2011; Olivera-Martinez et al., 2012), a signal which can repress FGF signalling and cell division, and thus promote cell differentiation (Abu-Abed et al., 2003; Diez del Corral et al., 2003; Olivera-Martinez et al., 2012). Differentiation of resident progenitors results in their exit from the tailbud, therefore, disruption of the balance between self-renewal and differentiation into mesodermal or neural fates can lead to the end of axial elongation. Additional mechanisms regulating resident behaviour and self-renewal may include changes in Lin28a and Wnt3a as dysregulation of these genes in mice has been shown to affect tailbud development and axis length (Aires et al., 2019; Greco et al., 1996; Robinton et al., 2019; Shum et al., 1999; Takada et al., 1994).

### 6.2 Cessation of division of axial stem cells

One alternative possibility to changes in local signalling is that resident axial stem cells undergo a pre-programmed number of divisions throughout axial elongation; once these are complete, the cells would leave the node and differentiate. Such pre-programming has been proposed for cells in the anterior streak, for which heterochronic grafts of anterior streak into the same position of younger hosts suggest that axial Hox identity is pre-set and not determined by the environment (Iimura and Pourquié, 2006). However, heterochronic grafts of tailbud regions into younger hosts suggest that unlike the streak, these graft-derived cells are able to reset their axial Hox signature (as shown for HOXA10, HOXC10 and HOXC8) (McGrew et al., 2008). Further evidence that resident cells respond to local signals rather than being pre-programmed comes from serial transplantation of tailbud sub-regions where the CNH repeatedly contributes both to cells with resident behaviour and to axial structures (Cambray and Wilson, 2002; McGrew et al., 2008). It is therefore unlikely that stem cells in the regressing node and tailbud are pre-programmed to divide a fixed number of times. However, it is possible that rather than individual cells being pre-programmed for a fixed number of cell divisions, cells of the tailbud might be pre-programmed to reduce FGF signalling gradually, thereby changing the tailbud environment.

### 6.3 Death of resident axial stem cells

Cell death has been assessed by the presence of pyknotic cells, where chromosome condensations that occur during cell death can be visualised and distinguished from live neighbouring cells, or by TUNEL staining to reveal apoptotic cells. An accumulation of cells undergoing cell death within the regressing node/tailbud has been observed from ∼HH14 in chick (Hirata and Hall, 2004). At HH17 pyknotic cells are observed in the TBM and the CNH of the tailbud (Sanders et al., 1986). By HH21/22, cell death extends further rostral, with pyknotic cells observed in tissues from tailbud up to the caudalmost somites, including pre-somitic mesoderm, notochord, neural tube and surface ectoderm (Mills and Bellairs, 1989; Sanders et al., 1986). By HH25-27, cell death is once again restricted to the tip of the tail (Mills and Bellairs, 1989; Schoenwolf, 1981). The highest level of cell death at ​∼ ​HH22 therefore coincides with the end of segmentation, with the stage when FGF expression begins to decline in the tailbud, and when retinoic acid begins to be synthesized within the tailbud via RALDH2 (Cunningham et al., 2011; Olivera-Martinez et al., 2012). However, it is unclear whether cell death is a cause or consequence of the changes in FGF and retinoic acid signalling observed. Although retinoic acid has been implicated in increased apoptosis of tailbud cells (Shum et al., 1999), RALDH2 is not necessary to terminate axial growth (Cunningham et al., 2011). Truncated mouse mutants such as the *Cyp26a1* homozygote null mutant, where retinoic acid is no longer catabolised in the regressing node/tailbud, do not result in an increase in cell death in the tailbud when checked at ​∼ ​E9.5–10.5 (Abu-Abed et al., 2001). Therefore, the link between an increase in retinoic acid and cell death within the tailbud in mouse is unclear. Cell death is therefore most likely initiated by other events/signals. A candidate gene involved in the regulation of cell death is HOXB13, which, when knocked out in mice, results in longer tails with increased cell proliferation and reduced cell death in the caudal spinal cord (Economides et al., 2003); conversely, when overexpressed, it instead reduces cell proliferation and increases apoptosis (Aires et al., 2019).

## 7 Conclusions

During head-to-tail development in amniotes, cells are progressively added to the developing axis from the node and tailbud. Single cell labelling experiments reveal that resident cells in the node can self-renew and contribute to the developing axial midline, consistent with stem-cell behaviour (Forlani et al., 2003; Lawson et al., 1991; Selleck and Stern, 1991). Furthermore, single cell transplantations show that the node can impart such stem cell behaviour on cells not normally fated to enter the node (Solovieva et al., 2022). The node therefore contains stem cells and is able to impart self-renewal on cells that enter it, thus acting like an instructive stem cell niche.

Fate mapping of node sub-regions suggests that the niche for self-renewal is located in the caudal part of the node (Cambray and Wilson, 2007; Solovieva et al., 2022; Wymeersch et al., 2019). Dividing resident cells in the caudal region of the node are sparse (Solovieva et al., 2022). Along with estimates of progenitor numbers in the node based on cell-cycle length and number of cells along the axis, it is likely that resident stem cells make up a small population relative to the size of the node (Selleck and Stern, 1991, 1992; Stern, 1996).

While resident cells have been identified at tailbud stages, this population is likely to have changed in composition from the resident stem cells in the younger node. Retrospective clonal analysis suggests that the tailbud might gain new cells from caudal lateral epiblast and lose some stem cells to differentiation (Brown and Storey, 2000; Tzouanacou et al., 2009). However, prospective single cell fate mapping for the entirety of axial elongation is required to confirm such hypotheses. The ability of the putative niche to impart self-renewing behaviour during tailbud stages is also yet to be confirmed. At the end of axial elongation, possible cellular mechanisms for the loss of stem cells in the tailbud include their exit from the tailbud through their differentiation and loss of resident behaviour (Mathis et al., 2001; Olivera-Martinez et al., 2012), or by their death (Mills and Bellairs, 1989; Sanders et al., 1986).

In summary, the node acts as an instructive stem cell niche in the early stages of axial elongation. This paves the way to future detailed characterization of this novel stem cell niche in development.
